# Thoughts on Medical Theories. No. 2

**Published:** 1854-01

**Authors:** 


### ART. VI.—*Thoughts on Medical Theories. No.* 2.

By Rusticus.

Messrs. Editors,—In some old Latin text book is written a bit of hepatic pathology, "*In jecore est officium fellis*," which some neophyte in Latinity translated, "in Jericho is the workshop of cats." This was a somewhat free translation, but it is not the only free rendering given to the functions of the liver.

While our entire knowledge of the liver could be summed up in the meagre statement that it was an organ located in the right hypochondrium, which secreted a fluid called bile, it was a very favorite locality for hypothesis. I have long since come to the conclusion, that it is most easy to talk learnedly on subjects which nobody understands. Many men have written deeply on mental philosophy, and from some essays that-wise, my own experience teaches me that it is a very easy matter to theorize about, so long as one maintains a comfortable ignorance as to certain ugly and contradictory facts; which do not gear in well with hypothesis. Thus it was with our hepatic pathologists. The more confused a man's knowledge of the anatomy and physiology of that organ, the more learnedly could he talk about it, "putting on the livery of science like a monkey dressed in regimentals."

It was a singular circumstance that the liver, which was so often diseased *during life*, should so uniformly present a healthy *post-mortem* appearance. But your true theorist never stumbles at facts. When we start with a theory, we see facts "through a glass darkly"—a smoked glass which sometimes smuts our noses. In this latter day M. Bernard has made sad havoc with hepatic pathology. We talk now of M. Bernard's *theory*. But I do not like the term. It is better to say his *facts*. He has no theory about the matter; he shows, by chemical analysis, that what goes into the liver as starch, comes out as sugar; that the fatty emulsion which the pancreas has prepared, goes unchanged into the liver to be there saponified, and rendered assimilable. He brings it down to a matter of organic chemistry. Jericho is no longer the workshop of cats, it is a sugar house.

People blunder upon truth sometimes, and our juvenile Latinist was an unconscious satirist. I am disposed to see a special providence in that translation—"Jericho." When we knew not what ailed a man, we referred the trouble to the liver; in a word, we sent all puzzling facts to Jericho.

All this talk about the liver is only a sort of opening illustration to my notions of theory. *A theory is a bridge of reasoning placed between two facts, or sets of facts, connecting them together. Ergo*, mine Editors, if you have not facts to start from, you can have no *true* theory. And there lies the pith of my objection to medical theory. We must have a perfect physiology, before we can have any basis for theoretical reasoning. We must have *all* the facts. I have no hope of living to see that day.

There is another consideration in this connection which is consolatory. I claim the preemption right to a new discovery which I believe to be a *fact*, and that is, that when we get a theory, or a set of principles in medicine, no matter how true they are, they will do us less good than we anticipate. I can understand the longing of the older physicians for a theory. I can feel within me the fire of enthusiasm which dictated their labors. I can appreciate, how, wearied of the cumbrous pile of facts which lay before .them, defying all order or arrangement, they should wish for some guiding principle—a touchstone which should test their value—a shibboleth, at the utterance of which they should fall into rank, a disciplined army ready for the war against disease. Thus they toiled for that which was an apple of Sodom in their grasp.

"*Eheu! quantus, ****** quantus erat sudor viris!*" Suppose that the dreams of theorists were realized—that from all the conflicting testimony, the myriad of facts, the annals of all recorded experience, were framed a theory brief, simple, comprehensive—a great first principle, which should govern the treatment of all disease. Even then we should have trouble in applying our principle. Divine Wisdom can enunciate, but human strength cannot profit by such a guide. The Sermon on the Mount contains the whole secret of happiness in life, and safety in death—the Golden Rule is the brief unfailing index for all our actions—the *theory* of Christianity is as perfect as the God who made it; but men live unhappily, cheat their neighbors, and Heathendom covers three-quarters of the world. All things must come at last to the judgment of the individual practitioner, and when we have a Golden Rule of medicine, we shall halt between two opinions, as to what is right, and what is wrong.

To come back to medical theories. Probably no one of these is more tangible, or promising, than that of the unity of disease. It is argued that all disease is in some one essential feature the same. This unity, (as one remarks who has written learnedly upon it,) is not an unity of organ, as Broussais would have it, nor of symptoms, nor of outward appearance, nor of treatment, but of pathology—and that pathology is congestion. That is, in disease, nervous, inflammatory, or poisonous, congestion is the one ever present phenomenon. Admitting this to be true, I ask "*cui bono?*" Shall we not still bleed a pneumonia, and stimulate a typhus?

Or we will suppose, (though that *is* an effort of the imagination!) that Mrs. Willard's theory of the circulation is true, to what practical purpose can we apply it? A sister schoolm— educator—of Mrs. Willard's, attempted its application, once upon a time, with a patient dying of cholera. She trundled the bed of the dying man to the open window, and implored him to keep breathing; "do make an effort!" said she. If he could only keep breathing the heart would keep beating, and if the heart beat, why of course he would not die. But he did die, and so will everybody else who depends on theory for life. * * * It is pleasant to find in Mrs. Willard's theory an old acquaintance—to recognize in it an idea as ancient as its real author, Dr. Malachia Thruston, who published at London, in the year 1670, "*De Respirationis Usu Primario Diatriba.*" The Dr. Cartwright of that day was Will. Musgrave, M. D., who, having no alligators, experimented on dogs. I omit the experiment, but refer for its analogue to the New Orleans or Boston Medical Journals, of a year or more ago. But here is Dr. Musgrave's *sequitur:* "This Experiment proves, that *Respiration* promotes the Passage of the Blood through the *Lungs;* and in Bodies full of vigorous Blood, it is, on this Account, of perpetual Necessity. This Acceleration of the Blood in that Passage, seems to be the principal Use of *Respiration;* no other is of such Consequence to Life, or stands in Competition with it."

The Hindoo mythology of the transposition of souls holds good in medical theories. They are perpetually reappearing on the stage of human action. This old notion was killed, almost two centuries ago, by one whose name stands mysteriously on the title page, as "Auth. *Georgio Entio* Equ. Aur., M. D., *Lond.*, 1671, in 8o." I recommend the republication of the learned knight's "autadiatriba," as apropos to the discussion now progressing.

There is something very funny in the solemn owlishness with which a new-old theory is propounded. One cannot help laughing at their odd appearance, and although it is very wrong to sneer at error in decrepitude, I am irresistibly reminded of the lines of the poet-physician concerning one who had outlived the fashions of his times:

"I know it is a sin
For me to sit and grin
At him here;
But the old three-cornered hat,
And the breeches, and all that
Are so queer."—*O. W. Holmes, M. D.*

It is growing late in the night, mine Editors. I have had a long ramble among the "black-letter lore" in search of Mrs. Willard's theory, since I first took up the pen. So good morning, for we are in the short hours.

